# The Development of Intergroup Cooperation: Children Show Impartial Fairness and Biased Care

**DOI:** 10.3389/fpsyg.2022.825987

**Published:** 2022-03-18

**Authors:** John Corbit, Hayley MacDougall, Stef Hartlin, Chris Moore

**Affiliations:** Department of Psychology and Neuroscience, Dalhousie University, Halifax, NS, Canada

**Keywords:** in-group bias, fairness, care, prosocial behavior, cooperation

## Abstract

One of the most remarkable features of human societies is our ability to cooperate with each other. However, the benefits of cooperation are not extended to everyone. Indeed, another hallmark of human societies is a division between us and them. Favoritism toward members of our group can result in a loss of empathy and greater tolerance of harm toward those outside our group. The current study sought to investigate how in-group bias impacts the developmental emergence of concerns for fairness and care. We investigated the impact of in-group bias on decisions related to care and fairness in children (*N* = 95; ages 4–9). Participants made decisions about how to allocate resources between themselves and a peer who was either an in-group or out-group member. In decisions related to care, participants were given two trial types on which they could decide whether to give or throw away a positive or negative resource. In decisions related to fairness participants and peer partners each received one candy and participants decided whether to allocate or throw away an extra candy. If the extra candy was distributed it would place either the participant or their recipient at a relative advantage, whereas if the extra candy was thrown away the distribution would be equal. We found that on fairness trials children’s tendency to allocate resources was similar toward in-group and out-group recipients. Furthermore, children’s tendency to allocate resources changed with age such that younger participants were more likely to allocate extra candies to themselves, whereas older participants were more likely to allocate extra candies to their recipient. On trials related to care we did observe evidence of in-group bias. While distribution of positive resources was greater than negative resources for both in-group and out-group recipients, participants distributed negative resources to out-group recipients more often compared to in-group recipients, a tendency that was heightened for young boys. This pattern of results suggests that fairness and care develop along distinct pathways with independent motivational supports.

## Introduction

One of the most striking features of human societies is the propensity to cooperate with others yet, the benefits of cooperation are not extended to everyone. In-group favoritism based on gender, religious, racial, or ethnic group identity can result in a loss of empathy and greater tolerance of harm toward out-group members and has been linked to differential health outcomes and access to resources (Christie and Allport, 1954; Cikara et al., 2011; Ridgeway, 2011). Indeed, while adults are often motivated to alleviate suffering and help their in-group, they show a strong reduction in care about the suffering of their out-group (for a review Xu et al., 2009; Cikara et al., 2011). Indeed, this loss of empathy toward out-group members even extends into antipathy (Brewer, 1999; Cikara et al., 2014). Beyond a loss of care for out-group members, adults show favoritism toward in-group members in their concern for fairness. For instance, adults allocate more resources to in-group members (Balliet et al., 2014), are more likely to punish inequality that is perpetrated by an out-group member (e.g., Bernhard et al., 2006; Baumgartner et al., 2011; Schiller et al., 2014) and enforce harsher punishments when inequality disadvantages in-group as opposed to out-group members (Bernhard et al., 2006). Together this pattern of findings suggests that in-group bias is a key determinant of moral behaviors in the domains of care and fairness.

Care about the wellbeing of others and fairness as a standard of justice are two concerns that are foundations across many theories of moral psychology (Turiel, 1983; de Waal, 1996; Smetana, 2006; Graham et al., 2011; Tomasello and Vaish, 2013). Care is driven by a desire that the needs of others are met. This concern is manifested behaviorally through prosocial behaviors such as helping, sharing and comforting (Dunfield et al., 2011). Care also has a reciprocal concern for the alleviation of others’ suffering, thus taking actions such as comforting to alleviate suffering and avoiding actions that may cause suffering (Graham et al., 2011; Paulus et al., 2020; Geraci et al., 2021). Fairness is a standard of justice through which outcomes between individuals are evaluated; equilibrium is met by balancing the perspectives of all stakeholders and arriving at mutually satisfactory outcomes (Piaget, 1997). Fairness is highly context dependent, but within distributive justice guiding principles include equality, equity, and need (Deutsch, 1975). Behaviors that maintain fairness in the context of distributive justice include allocating resources according to principles of fairness, punishment of fairness violations, and rejecting distributions that are unfair (Fehr and Schmidt, 1999; McAuliffe et al., 2017a).

Like adults, children show in-group favoritism in behaviors related to care and fairness emerging in the preschool years (Over, 2018). Between 3- and 5 years of age, children show greater generosity to race-matched peers (Zinser et al., 1981; Renno and Shutts, 2015) and also share more with gender-matched peers (Dunham et al., 2011, 2016; Renno and Shutts, 2015). Although younger children (5–6 years of age) show a preference for their racial in-group, older children (6–11 years of age) appear to overcome this bias in favor of equity across groups (Olson et al., 2011; Elenbaas and Killen, 2016; Elenbaas et al., 2016; Rizzo et al., 2018), suggesting that principles of equity may overcome in-group bias as children move into middle childhood.

Children’s in-group bias also occurs in minimal group contexts, an experimental manipulation in which group status is assigned arbitrarily (often based on T-shirt color), thereby parsing in-group bias from prejudice directed toward specific social categories (Tajfel, 1970). For instance, children aged 3–6 years tend to be more generous toward in-group members (Sparks et al., 2017), a pattern that appears stronger amongst young boys (Benozio and Diesendruck, 2015). Children from Canada and Iran between 5 and 6 years of age are more likely to choose the equal allocations over an advantageous allocation when their recipient was an in-group member compared to an out-group recipient (Keshvari et al., 2021). In the same study when children chose between equal and disadvantageous allocations the rate at which they chose equal allocations was not influenced by recipient group. In studies that have explicitly probed the influence of in-group favoritism on fairness concerns we see a mixed pattern of results. As third party observers, children aged 6–8 years are more likely to punish selfish behavior perpetrated by out-group members, particularly when in-group members are harmed (Jordan et al., 2014). However, when children were themselves the recipients of unequal distributions of resources, they do not appear to show in-group bias in punishment or in rejection of inequality (McAuliffe and Dunham, 2017; Gonzalez et al., 2020).

Much like adults, children’s favoritism toward in-group members is coupled with a loss of empathy and greater tolerance of harm toward out-group members (Aboud, 2003; Cikara et al., 2014; Kteily et al., 2016; McLoughlin and Over, 2018). For example, 3- to 6-year-old children who learned about the preferences of their recipients tended to give preferred resources to in-group members, yet boys were also more likely to give items that were disliked to out-group members (Benozio and Diesendruck, 2015). Similarly, 6- to 8-year-old children gave more positive resources to in-group members, while only 8-year-old children gave more negative resources to out-group members, a tendency that was once again stronger for boys than girls (Buttlemann and Böhm, 2014). Other studies have examined the influence of in-group bias on helping, another behavior related to care, showing that children (5–10 years of age) are more willing to provide help to racial in-group members (Katz et al., 1976), a preference that extends to minimal groups amongst 5 years old children (Plötner et al., 2015).

Thus, when examining the development of in-group bias on the moral domains of care and fairness we are met with a complex picture. In contexts related to care children often show favoritism to their group, yet fairness results are mixed. Across these studies, in-group favoritism has been contrasted with either fairness concerns or care, but we are not aware of research where these concerns have been evaluated simultaneously, using a methodology that is able to specifically parse children’s concern for fairness and their concern of care. By examining the impact of in-group bias on fairness and care within the same children we could gain important insight into the relative impact of in-group bias on these two domains and examine developmental changes in these preferences.

The current study sought to investigate how in-group bias impacts the emergence of concerns for fairness and care. We assigned children between 4 and 9 years of age to groups using a minimal group technique (Dunham et al., 2011). Participants were then presented with a resource allocation task wherein a hypothetical peer was identified as either an in-group or out-group member who would be the recipient of participant’s allocation decisions. The decision that participants were given in the resource allocation task was always whether to give or throw away a resource. To investigate the influence of in-group bias on Care, participants were given two trial types on which they could decide whether to give or throw away resources that had been identified by the partner as having a positive or negative valence, a preferred animal sticker or an aversive spider sticker (adapted from Buttlemann and Böhm, 2014). Specifically, in this task Care would be exhibited by giving positive resources and throwing away negative resources as it would show a sensitivity to the desires of the recipient. To investigate fairness concerns, participants and their recipients each received one candy and participants had to decide whether to allocate or throw away an extra candy (adapted from Shaw and Olson, 2012). We presented two trial types designed to elicit two forms of inequity aversion; on advantageous trials participants could keep the extra resource or throw it away (a measure of advantageous inequity aversion–AI), on disadvantageous trials they could give it to the recipient or throw it away (disadvantageous inequity aversion–DI). We employed a fully within subject design where participants were presented with each trial type in a resource allocation task across two blocks of 12 trials. In one block the recipient was an in-group peer and in the other an out-group peer.

We hypothesized that in-group bias would be more likely to emerge in the domain of care relative to fairness. Empathy is an important foundation for care but perhaps not fairness (Decety and Cowell, 2015), and empathy has been found to be stronger between in-group members compared to out-group members (Cikara et al., 2011, 2014). Further, based on previous findings we predicted that the in-group bias in care would be stronger for young boys than girls (Buttlemann and Böhm, 2014; Benozio and Diesendruck, 2015). We were not able to make specific predictions on the developmental trends for the effect of in-group bias on care as findings are mixed as to whether in-group bias increases (Buttlemann and Böhm, 2014) or decreases (Olson et al., 2011; Elenbaas et al., 2016; Rizzo et al., 2018) with age in the domain of care. In contrast to care, fairness concerns may override in-group bias. Fairness is hypothesized to depend largely on cooperative norms (Fehr and Fischbacher, 2004) and prominent theories have argued that fairness is founded upon a concern for treating others impartially and with respect (Shaw and Olson, 2014; Engelmann and Tomasello, 2019). Thus, in line with previous work (Gonzalez et al., 2020) we predicted that fairness concerns would be applied impartially, especially amongst older children (7–9 years of age) who tend to show a strong concern for equity-based fairness (Shaw and Olson, 2012; Blake et al., 2015).

## Materials and Methods

### Participants

We sampled 95 participants (*n* = 52 girls) between the ages of 4 and 9 years (*M* = 7.12, SD = 1.82). Participants were sampled with the goal of balancing across age and gender (see Supplementary Table 1). One participant was excluded due to experimenter error. We chose this sample size based on typical samples in prior work examining the development of in-group bias on resource allocation decisions (Buttlemann and Böhm, 2014; Sparks et al., 2017). Participants were recruited through the participant database of the Early Social Development Lab (ESDL) at Dalhousie University, Halifax, NS, Canada, and our sample was one of convenience. Parental consent was obtained prior to the session and child assent was obtained at the beginning of each session. This research was approved as minimal risk by the Research Ethics Board at Dalhousie University (file #2020-5308).

### Materials

Each child was assigned to either a red or green team by picking a red or green coin by chance. Participants were then given team T-shirt that corresponded to the color of the team (green or red) that they were assigned to. Photographs of four children (two boys and two girls) were used to depict the recipients in the sharing task. Children in the photographs appeared to be similar of age to the participants. All four children were depicted in two photographs, once wearing a green uniform and once wearing a red uniform, so that each recipient could be randomly assigned to either the in-group or out-group.

#### Resource Allocation Task

On Fairness trials we used commonly available candies (Skittles) as the resource. On Care trials children were given 3D stickers that depicted spiders (for harm trials) and animals (for care trials). We used small paper bags for the participant to put resources for themselves and for the recipient and a toy trash can for the resources the participant wanted to throw away.

### Procedure

The procedure began with a minimal group induction with the participant randomly assigned to one of two “teams” based on green or red T-shirt color (adapted from Dunham et al., 2011). The induction began as the researcher presented the participant two coins (green and red) corresponding to a green team and a red team. The researcher placed these coins in their hands and hid them behind their back, then asked the participant to point to one of her arms. The coin in the chosen hand determined which T-shirt color the participant was assigned. The researcher gave the participant their T-shirt to wear, then presented the participant with two pictures: one of children wearing green T-shirts and the other of children wearing red T-shirts. A comprehension check was conducted where the researcher asked the participant which picture showed their team to ensure recognition of group membership and all participants identified their group correctly without further prompting.

The researcher introduced participants to a picture of a gender-matched peer recipient, described as a real individual who would “play the game later.” This recipient was either an in-group or out-group member, which was assigned randomly prior to testing. The researcher showed participants a paper bag attached to the recipient’s picture for any resources they wanted to give the recipient and another paper bag for any resources the participant wanted to give themselves. The researcher also explained that the “trash can” was for any resources the participant wanted to throw away and not give to anyone.

Next, the researcher introduced the resources used in the trials. For fairness trials we used candies, and for care trials we used spider stickers and animal stickers. The researcher told the participant that the recipient liked candy and asked the participant if they also liked candy, recording this response on the coding sheet. Further, the researcher told the participant that the recipient liked animal stickers (positive resource) but did not like spider stickers (negative resource). In this task Care for the recipient would manifest in giving a positive resource and throwing away a negative resource. A comprehension check was done to ensure participants understood what the recipient liked and disliked and all participants answered the questions correctly. Children were then given 12 trials, three each of the four trial types (see Supplementary Table 2). The order of trials was randomized. For the second block, researchers switched pictures to a gender-matched peer with the opposite shirt color as the first picture and introduced the participant to the new recipient. The researcher reintroduced the bags, trash can, and resources, stating the same likes and dislikes as for the first recipient. After a second comprehension check on the recipient’s preferences, we administered 12 additional trials. Group membership (in-group and out-group) of the first and second recipients was counterbalanced across participants.

#### Fairness Trials

Two of the four trial types were relevant to fairness and used a method adapted from Shaw et al. (2016). These trials entailed a choice between a fair or unfair distribution and assessed participants’ allocation decisions. The participant was presented with a distribution creating advantageous inequity (AI) in one trial type and disadvantageous inequity (DI) in the other. AI trials allowed the participant to choose between distributing an extra resource to themselves or throwing it away to achieve equity. DI trials allowed the participant to choose between distributing an extra resource to the recipient or throwing it away to achieve equity. For these trials, researchers placed one candy in front of the participant and one in front of the recipient’s picture, then showed the participant one extra candy. In AI trials, the participant was asked if they wanted to give this extra candy to themselves or throw it away. In DI trials, the participant was asked if they wanted to give this extra candy to the recipient or throw it away. There were three AI and three DI trials for both the in-group and out-group conditions, totaling 12 fairness trials per participant.

#### Care Trials

Two of the four trial types were relevant to care and used a method adapted from Buttlemann and Böhm (2014). The resources used in these trials either had positive valence or negative valence, as established with the participant prior to the trials. Positive valence resources were animal stickers, which the researcher explained that the recipient liked. Negative valence resources were spider stickers, which the researcher explained that the recipient disliked. The researcher placed one sticker in front of the participant and asked if they would like to distribute this sticker to the recipient or throw it away. There were three trials consisting of positive resource allocation (animal stickers) and three trials consisting of negative resource allocation (spider stickers) for both the in-group and out-group conditions, totaling 12 care trials per participant.

After the experimental trials the researcher asked the participant if they would prefer to play with the recipient on the green team or the red team in order to gain a convergent measure of in-group preference. The researcher also assessed the participant’s own preference for spider or animal stickers following experimental trials. Amongst participants who expressed a sticker preference (*n* = 78), the majority of both males (*n* = 23) and females (*n* = 30) preferred animal stickers, while some males (*n* = 14) and females (*n* = 11) expressed a personal preference for the spider stickers.

### Data Coding and Analyses

All sessions were videotaped. The primary outcome variable was the number of trials on which children chose to give (coded as 1) or throw away (coded as 0) a resource in the resource allocation task. Children’s decisions were recorded live, and reliability checked from video by a video coder who was blind to the study hypotheses. Disagreements between the live and video coding were rare (Cohen’s κ = 0.95) and were resolved by rechecking the trials from video.

In order to investigate whether the likelihood that children choose to give resources was influenced by our test predictors: Age (continuous), Distribution (positive, negative, advantageous, and disadvantageous), Group (in-group or out-group) and their interactions, we used a Generalized Linear Mixed Model (GLMMs; Bolker et al., 2009) with binomial error distribution and logit link function. In preliminary analyses we conducted a test to see if Trial Type (Fairness and Care) was a significant predictor of children’s allocation decisions and found that the overall rate of giving was influenced by Trial Type (LRT: χ^2^ = 75.64, df = 4, *p* < 0.001). Thus, the analyses of participants’ allocation decisions were performed separately for each Trial Type.

Our first step in data analysis was to build full models for both fairness and care trials that included the three-way interaction between Age, Distribution (advantageous and disadvantageous or positive and negative), and Group (in-group and out-group). Participant identity (ID) was fit as a random effect (intercepts) to control for repeated measures and participant gender was included as a control effect. The models were fitted in R using the function “glmer” from the R package “lme4” (Bates et al., 2012). All figures were created in R and were made using the package “ggplot2” (Wickham, 2009). The statistical significance of the full model was determined by comparing its fit with that of the null model comprising only the random effect, using a likelihood ratio test (LRT), available as R function “anova,” package “stats.” *p*-Values for individual effects were based on LRTs comparing the full models with their respective reduced models (R function “drop1”). The LRT was used for testing the interactions for significance, and non-significant interactions were removed from the model to reliably interpret the lower terms.

Gender has been shown to influence children’s in-group bias in resource allocation tasks, with males showing a greater tendency toward in-group bias in the domain of care (Buttlemann and Böhm, 2014; Benozio and Diesendruck, 2015). Thus, a second step in our data analysis plan was to investigate whether the relation between our test predictors: Age, Distribution, and Group varied by gender. The addition of Gender to the full model resulted in a significantly better fit to the data (LRT: χ^2^ = 38.48, df = 8, *p* < 0.001), thus children’s behavior on care trials was analyzed separately by gender.

## Results

### Fairness

**FIGURE 1:**
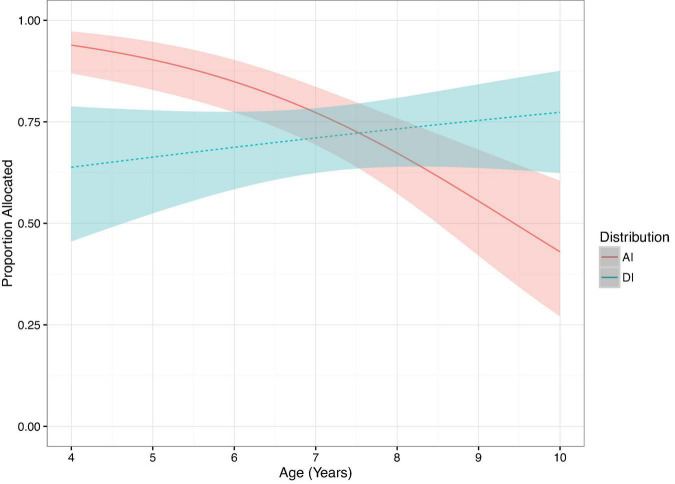
Predicted proportion allocated on Fairness trials by Distribution, plotted over age. Ribbons show 95% confidence intervals.

The comparison of the full against the null model was significant (LRT: χ^2^ = 56.91, df = 7, *p* < 0.001). The three-way interaction between Age × Distribution × Group was not significant (LRT: χ^2^ = 0.05, df = 1, *p* = 0.82; Supplementary Figure 1). The model was reduced by dropping all non-significant two-way interactions included in the three-way interaction (Group × Age, LRT: χ^2^ = 0.20, df = 1, *p* = 0.66; Distribution × Group, LRT: χ^2^ = 0.15, df = 1, *p* = 0.70). We further reduced the model by dropping the non-significant main effect of Group (LRT: χ^2^ = 0.45, df = 1, *p* = 0.50) and Gender (LRT: χ^2^ = 0.06, df = 1, *p* = 0.80). The final model was comprised from the significant two-way interaction between Distribution and Age (LRT: χ^2^ = 50.57, df = 1, *p* < 0.001, Figure 1). Between 4 and 9 years of age, participants decreased the allocation of candies on advantageous trials (LRT: χ^2^ = 12.32, df = 1, *p* < 0.001), yet the tendency to allocate candies remained stable on disadvantageous trials (LRT: χ^2^ = 1.02, df = 1, *p* = 0.32). Figure 1 reveals that younger participants were more likely to allocate extra candies to themselves, whereas older participants were more likely to allocate extra candies to their recipient.

### Care

**FIGURE 2:**
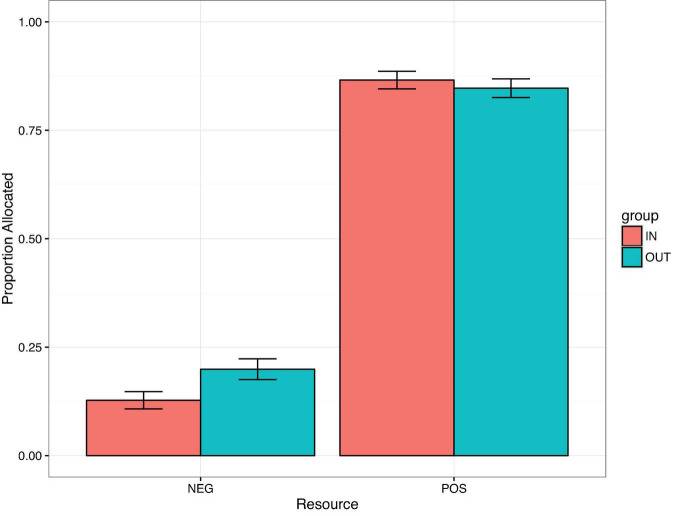
Proportion allocated on Care trials by Distribution and Group. Error bars show binomial confidence intervals.

The comparison of the full against the null model was significant (LRT: χ^2^ = 658.59, df = 7, *p* < 0.001) for care trials. The three-way interaction between Age × Distribution × Group was not significant (LRT: χ^2^ = 0.60, df = 1, *p* = 0.44; Supplementary Figure 2). The model was reduced by dropping the non-significant two-way interaction included in the three-way interaction, (Group × Age, LRT: χ^2^ = 0.24, df = 1, *p* = 0.62). The final model was comprised of the significant two-way interactions between Distribution and Age (LRT: χ^2^ = 9.75, df = 1, *p* < 0.01) and Group and Distribution (LRT: χ^2^ = 4.95, df = 1, *p* = 0.026, Figure 2) and a significant main effect of Gender (LRT: χ^2^ = 4.23, df = 1, *p* = 0.040). *Post hoc* analyses (mvt corrected) revealed that participants were more likely to give negative resources to out-group compared to in-group recipients (β = 2.41, *p* = 0.016) but no such difference was observed for positive resources (β = 0.75, *p* = 0.45). Figure 2 reveals that distribution of positive resources was greater than negative resources for both in-group and out-group recipients, however participants distributed negative resources to out-group recipients more often compared to in-group recipients.

**FIGURE 3:**
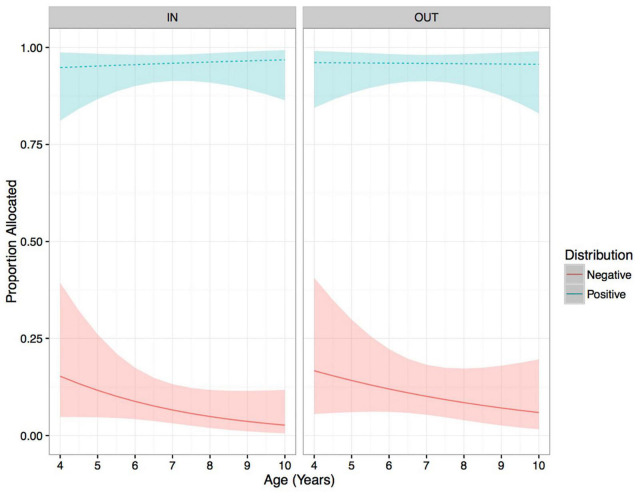
Predicted proportion allocated for females on Care trials, Distribution, facetted by Group, plotted over age. Ribbons show 95% confidence intervals.

**FIGURE 4:**
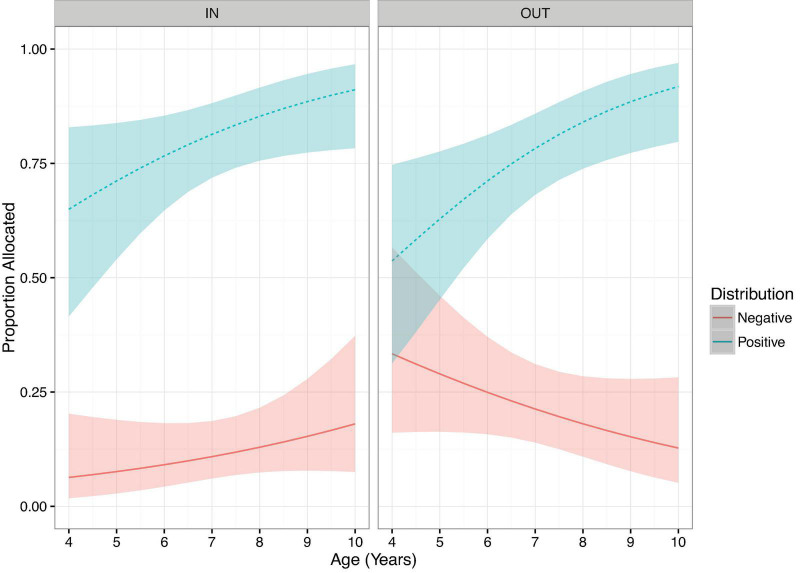
Predicted proportion allocated for males on Care trials, Distribution facetted by Group, plotted over age. Ribbons show 95% confidence intervals.

Gender has been shown to influence children’s in-group bias in resource allocation tasks, with males showing a greater tendency toward in-group bias in the domain of care (Buttlemann and Böhm, 2014; Benozio and Diesendruck, 2015). Thus, we examined the influence of the three-way interactions between Age × Distribution × Group for females and males separately. For females the three-way interaction was not significant (*p* = *0.51*, Figure 3), nor were any two-way interactions (all *p* > 0.1). The only significant predictor of female participants’ allocations was a main effect of Distribution (LRT: χ^2^ = 472.12, df = 1, *p* < 0.001), with significantly more giving of positive resources compared to negative resources (β = 13.07, *p* < 0.001). In contrast, for males the three-way interaction was marginally significant (LRT: χ^2^ = 3.37, df = 3, *p* = 0.066, Figure 4). This marginal three-way interaction tentatively suggests that young males allocated positive and negative resources at a similar rate for out-group recipients, whereas older male participants were much more likely to allocate positive resources compared to negative ones. For in-group recipients, participants were more likely to allocate positive resources compared to negative ones across the age range. To further examine this trend, we conducted exploratory analysis where age was coded as a categorical variable (4–6 and 7–9 years of age), in this case the three-way interaction between Age Group × Distribution × Group was statistically significant (LRT: χ^2^ = 5.79, df = 1, *p* = 0.016). *Post hoc* analysis revealed that younger males (4–6 years) are more likely to give negative resources to out-group recipients compared to in-group recipients (β = 2.97, *p* = 0.003; no other contrasts approached the threshold for statistical significance).

Returning to our panned analysis with Age as a continuous variable, dropping the marginally significant three-way interaction from the model resulted in significant two-way interactions between Distribution and Age (LRT: χ^2^ = 9.01, df = 1, *p* < 0.01) and Group and Distribution (LRT: χ^2^ = 6.25, df = 1, *p* = 0.012). *Post hoc* analyses (mvt corrected) revealed that male participants were more likely to give negative resources to out-group compared to in-group recipients (β = 2.36, *p* = 0.018), but no such difference was observed for positive trials (β = 1.07, *p* = 0.28). This pattern suggests that the tendency to give more negative resources to out-group compared to in-group recipients was stronger for males compared to females.

## Discussion

Our primary goal in this study was to examine the development of in-group bias in children’s resource allocation decisions in the domains of care and fairness. On fairness trials we did not observe evidence of in-group bias on children’s allocation decisions, suggesting that fairness behavior was not influenced by in-group bias. Children’s decisions on fairness trials allowed us to examine the developmental emergence of their aversion to advantageous and disadvantageous inequity from early to middle childhood. We found that between 4 and 9 years of age children became less likely to give themselves a personal advantage. In contrast their tendency to allow their peer to gain an advantage remained stable across this age range.

In the domain of care, children were increasingly likely to allocate positive resources and less likely to allocate negative resources across age. In line with our hypotheses, we did observe evidence of in-group bias on care trials. Specifically, children were more likely to allocate negative resources to out-group than in-group recipients, however, no group effect was observed for positive resources. Finally, this tendency to allocate negative resources to out-group recipients was largely driven by young males, we did not observe evidence of in-group bias amongst older participants or amongst females participants.

On fairness trials we did not observe an effect of in-group bias on children’s fairness behavior. This pattern held for allocation decisions that placed the participants at either an advantage or disadvantage relative to their peer. In the current study advantageous trials provided a strong test of fairness, equal outcomes came at a cost to the participant and did not provide a material benefit to the recipient. In the case of disadvantageous trials equal outcomes incurred a cost to the recipient, thus may be motivated by spite or envy rather than fairness (Shaw and Olson, 2012; McAuliffe et al., 2014), whereas unequal outcomes provided a material benefit to the recipient and could be motivated by generosity. Several previous studies found that children were more likely to choose equal allocations over advantageous ones when recipients were in-group members (Sparks et al., 2017; Keshvari et al., 2021). In these studies, equal outcomes on advantageous trials provided a material benefit to the recipient, thus may have been motivated by generosity. However, increased generosity to in-group recipients does not account for behavior on disadvantageous trials where children’s tendency to provide their recipient with a relative advantage was not influenced by group status. Thus, the weight of the evidence across these studies favors an increased concern for fairness with in-group recipients (Sparks et al., 2017; Keshvari et al., 2021). Another study that examined the impact of in-group bias on children’s aversion to advantageous and disadvantageous inequity did not find strong evidence of in-group favoritism on children’s aversion to inequity, though the results were inconclusive in the case of advantageous inequity aversion (Gonzalez et al., 2020). In the current study we did not see evidence that in-group bias influenced either generosity or fairness, thus the precise impact that in-group bias has on the development of fairness remain an open question.

In contrast to fairness we did observe in-group bias on care trials. Empathy is purported to be a core process through which in-group bias can influence people’s tendency to care for in-group members and tolerate the harm of out-group members (Batson and Ahmad, 2009; Cikara et al., 2011; Kteily et al., 2016). Indeed, increased empathy toward in-group members is found even in a minimal group context (Masten et al., 2010). Importantly, empathy is more likely to influence care relative to fairness decisions (Hoffman, 1984; Eisenberg and Miller, 1987; Decety and Cowell, 2015), and may explain the different impact of group status across these domains. Characteristic of an empathy-based response, behaviors related to care have the signatures of an intrinsic motivation to benefit the wellbeing of others. During infancy, children prefer agents that provide help rather than harm (Hamlin et al., 2007). From 2 years of age toddlers show the physiological manifestations of relief when they see someone receiving help, even when they are not directly involved (Hepach et al., 2012), and an affective benefit of their own generosity (Aknin et al., 2012). Finally, from 3- to 6-years-of-age children’s generosity increases with their understanding of the affective benefits of sharing (Paulus and Moore, 2017).

Bolstering the argument that empathy and fairness are distinct processes is evidence showing that children’s concern for fairness is heavily influenced by cooperative norms and arises from a desire to signal fair behavior to others. For example, children (6–8 years of age) are less likely to behave fairly if they could appear fair to an adult experimenter but act selfishly (Shaw and Olson, 2014). Similarly, children (6–9 years of age) are more likely to avoid personally advantageous distributions of resources when a recipient is observing their decisions, compared to when their actions are not observed (McAuliffe et al., 2020). Convergent evidence suggests that adherence to cooperative norms is a related determinant of fairness behavior (McAuliffe et al., 2017b; House et al., 2020). Together, these findings reveal that appearing fair and adhering to norms of fairness are important extrinsic motivators related to fairness behavior. Overall, while fairness behaviors have several extrinsic motivators that operate independently of empathic responses, behaviors related to care appear to be dependent on an empathetic response.

### Limitations and Future Directions

In this study we sought to investigate the influence of in-group bias in children concerns for care and fairness. In this initial investigation we employed a minimal group paradigm to induce group membership, as we wanted to assess the role of in-group bias independently of social preferences toward specific social categories. Thus, it remains an important open question as to how children’s concern for care and fairness may differ toward recipients that vary in terms of social categories such as gender and race. Previous work suggests that young children’s resource allocation decisions show a preference for gender and race matched peers (Zinser et al., 1981; Dunham et al., 2011, 2016; Renno and Shutts, 2015), yet by middle childhood children are often willing to rectify group based inequality (Olson et al., 2011; Elenbaas and Killen, 2016; Elenbaas et al., 2016; Rizzo et al., 2018; Corbit et al., 2021). Future research that investigates the influence of social categories such as gender and race on children’s concern for both care and fairness will provide important insight into how group-based prejudice can influence cooperative behavior.

## Conclusion

Overall, our findings indicate that in-group bias differentially impacted children’s moral behavior in the domains of care and fairness. In the domain of fairness, decisions were similar for in-group and out-group recipients. In contrast, in the domain of care children were more likely to allocate negative resources to an out-group compared to an in-group peer, revealing a tendency toward out-group harm that was particularly pronounced amongst boys. This pattern of results suggests that fairness and care develop along distinct pathways with independent motivational supports.

## Data Availability Statement

The datasets presented in this study can be found in online repositories. The names of the repository/repositories and accession number(s) can be found below: https://osf.io/zyfma/?view_only=51d3375a740d4edfbdcb718ebd27c5b5.

## Ethics Statement

The studies involving human participants were reviewed and approved by the Research Ethics Board Dalhousie University. Written informed consent to participate in this study was provided by the participants’ legal guardian/next of kin.

## Author Contributions

JC and CM developed the study design and concept. HM and SH conducted the testing and data collection. JC analyzed and interpreted the findings. JC drafted the manuscript with CM and HM. SH provided the critical feedback. All authors contributed to the article and approved the submitted version.

## Conflict of Interest

The authors declare that the research was conducted in the absence of any commercial or financial relationships that could be construed as a potential conflict of interest.

## Publisher’s Note

All claims expressed in this article are solely those of the authors and do not necessarily represent those of their affiliated organizations, or those of the publisher, the editors and the reviewers. Any product that may be evaluated in this article, or claim that may be made by its manufacturer, is not guaranteed or endorsed by the publisher.
