# Supplementary material for: The Development of Intergroup Cooperation: Children Show Impartial Fairness and Biased Care
Source: Front Psychol. 2022 Mar 18;13:825987. doi: 10.3389/fpsyg.2022.825987 (PMC8974294; doi:10.3389/fpsyg.2022.825987)
Supplement: Supplementary file 1 [file Data_Sheet_1.docx]

**Supplementary online materials:** The development of intergroup cooperation: Children show impartial fairness and biased care

# Supplementary Figures and Tables

## Tables

**Table S1.** Distribution of participants separated by age and gender.

| Gender | Age Group | | | |
| --- | --- | --- | --- | --- |
|  |  | 4&5 | 6&7 | 8&9 |
|  | Male | 11 | 15 | 16 |
|  | Female | 18 | 13 | 21 |

**Table S2.** Distribution of trial categories (Fairness and Care), trial types (AI, DI, Positive, Negative) and number of trials per block.

| Category of Trial | | Fairness | | Care | |
| --- | --- | --- | --- | --- | --- |
|  | Trial Type | Advantageous  Inequity | Disadvantageous  Inequity | Positive | Negative |
|  | Number of trials | 3 | 3 | 3 | 3 |

## Figures

**Figure S1**. Predicted proportion allocated on Fairness trials, Distribution, facetted by Group, plotted over age. Ribbons show 95% confidence intervals.

**Figure S2.** Predicted proportion allocated on Care trials, Distribution, facetted by Group, plotted over age. Ribbons show 95% confidence intervals.
